# Toxic Epidermal Necrolysis, A Serious Side Effect of Tenoxicam Use: A Case Report

**DOI:** 10.3390/healthcare11152195

**Published:** 2023-08-03

**Authors:** Tiberiu Paul Neagu, Mirela Tiglis, Ileana Peride, Andrei Niculae

**Affiliations:** 1 Clinical Department No. 11, “Carol Davila” University of Medicine and Pharmacy, 050474 Bucharest, Romania; dr.neagupaul@gmail.com; 2 Department of Anesthesia and Intensive Care, Emergency Clinical Hospital of Bucharest, 014461 Bucharest, Romania; 3 Clinical Department No. 3, “Carol Davila” University of Medicine and Pharmacy, 020021 Bucharest, Romania; niculaeandrei@yahoo.com

**Keywords:** tenoxicam, selective COX-2 inhibitor, skin adverse reaction, toxic epidermal necrolysis, corticosteroids, skin biopsy

## Abstract

Tenoxicam, a selective cyclooxygenase (COX)-2 inhibitor, has potent analgesic and anti-inflammatory effects and is frequently used for out-of-hospital pain control. Even though other non-steroidal anti-inflammatory drugs were incriminated in Stevens–Johnson syndrome (SJS) and toxic epidermal necrolysis (TEN) appearance, the literature is scarce regarding this agent. We report a case of tenoxicam-induced toxic epidermal necrolysis, detailing the multidisciplinary approach in a patient presenting skin detachment of 90% of the total body surface area, with concomitant ocular, oral, nasal, and vaginal mucosae involvement. A skin biopsy confirmed the diagnosis. The immediate cessation of the incriminated drug and rapid initiation of systemic steroids, along with topical therapies, and isolation into a specific environmental condition to limit skin infection were the cornerstones of therapeutic management. The patient was discharged with skin hyperpigmentation area and mild anxiety as long-term sequels. This report emphasized that severe or complicated cases should be transferred to a specialized burn center to reduce mortality risk and long-term morbidity.

## 1. Introduction

Stevens–Johnson syndrome (SJS) and toxic epidermal necrolysis (TEN) are serious mucocutaneous reactions associated especially with drugs such as antibiotics (sulfonamides, trimethoprim-sulfamethoxazole, penicillin, macrolides, and fluoroquinolones), anticonvulsants (lamotrigine, phenobarbital, and carbamazepine), allopurinol, non-steroidal anti-inflammatory drugs (NSAIDs), corticosteroids, acetaminophen, and antiretrovirals [1,2,3,4]. There are recent reports about SJS/TEN development in association with anticancer agents (daunorubicin, alpelisib, fulvestrant, and enzalutamide) [5,6,7,8]; elastomeric pump use for chronic pain management in a patient with late-stage ovarian cancer, probably due to ondansetron [9]; armodafinil and modafinil [10,11]; chlordiazepoxide [12]; pirfenidone [13]; or after sulfadoxine–pyrimethamine overdose [14]. Lesions usually appear 4–28 days after the patient is exposed to the incriminating agent [15].

If there is less than 10% epidermal detachment, the diagnosis is SJS, with a mortality risk of 1–5%. If there is between 10–30% involvement of skin, the diagnosis is SJS-TEN overlap syndrome. The more severe TEN designation is used when epidermal detachment is greater than 30%. TEN has been observed to have a mortality rate of >40% [16,17]. The incidence is 0.4–6 per million people each year [18,19]. Patients with cancers and immunodeficiency, as well as women, are more vulnerable in developing adverse drug reactions [20]. A recent study by Gronich et al. identified additional risk factors including psoriasis, systemic lupus erythematosus, a history of cerebrovascular accident or diabetes mellitus, and previous drug allergies [21]. SJS and TEN are delayed-type hypersensitivity reactions to specific drug administrations; therefore, these reactions are considered medical emergencies that require rapid diagnosis and prompt therapeutic management [15]. Individuals with a genetic predisposition are more susceptible [22]. Some reports showed that there may be a connection between human leukocyte antigens (HLA)-B*12 and (HLA)-B*73:01 and oxicam-induced TEN in European populations [23,24].

Tenoxicam is an NSAIDs in the oxicam group used as a potent analgetic agent [25]. In addition, it has anti-inflammatory and antipyretic activity [26]. The most common side effects include gastrointestinal bleeding [27], skin rashes, and central or peripheral nervous system disorders [28,29]. However, rare cases of hepatic injury [30]; alopecia [31]; nephrotoxicity [32], especially in patients with pre-existing renal dysfunction and the elderly [33,34]; hypoglycemia [35]; and agranulocytosis were reported [36]. Nevertheless, there are concerns regarding the safety profile of cyclooxygenase (COX) inhibitors, especially in patients with cancer or immunosuppression and a history of hypersensitivity [37]. Ward et al. stated that even if the risk of SJS or TEN onset after NSAIDs is extremely low, there are some at-risk categories, like the elderly, women, and patients during the first month after the drug initiation, especially when oxicam derivates are used [38].

A French study targeting the adverse drug reactions associated with NSAID administration showed that the highest risks for significant gastrointestinal, hepatic, renal, or cutaneous adverse events were associated, in order of severity and frequency, with ketoprofen, nimesulide, meloxicam, and tenoxicam compared with the other NSAIDs [39]. There are reports about SJS or TEN appearances after selective cyclooxygenase (COX) inhibitor use, with valdecoxib, celecoxib, piroxicam, etoricoxib, and rofecoxib being usually incriminated [2,40,41,42]. Mockenhaupt et al. demonstrated that NSAID-induced SJS/TEN has low risk overall, with the oxicam class having a higher risk, especially during treatment initiation [43]. The EuroSCAR study also reported that the use of oxicam-NSAIDs (meloxicam, piroxicam, and tenoxicam) is associated with a high risk of severe cutaneous adverse reaction occurrence within 8 weeks of treatment and that these agents should be avoided as first-line therapies. It also emphasizes the importance of identifying the moment of drug administration for such cases [1].

Over the years, various cases of SJS/TEN have been reported in relation to analgesic drug utilization. Tenoxicam was found to be the causative agent for TEN/SJS in some studies, but there are no singular reported cases about this drug [1,39]. Friedman et al. reported a case of TEN appearance because of celecoxib use for carpal tunnel syndrome treatment. Due to the disease severity, the patient was transferred to a burn center, having a good long-term prognostic [44]. However, Perna et al. reported a fatal case of TEN after celecoxib use for lower back pain in a female patient [45]. Etoricoxib taken for pain-control in a female with osteoarthritis was involved in severe TEN appearance reported by Kameshwari et al., with a good outcome following glucocorticoid therapy as the main treatment [2]. A fatal case after etoricoxib use in a young female patient with a sprained ankle was presented by Roy et al., with rapid deterioration under corticosteroids [46]. Massari et al. reported a case of ketoprofen-induced TEN associated with acute vanishing bile duct syndrome [47], similarly to a case presented by Kim et al., but the causative agent for TEN in this patient was ibuprofen [48]. Ibuprofen was also incriminated in a female case of severe toxic epidermal necrolysis following its use for upper respiratory tract infection symptoms. Treatment with high-dose corticosteroids had good results [49]. Severe drug-induced TEN was also reported in relation to diclofenac sodium, with a favorable outcome following plasmapheresis use [50].

Frey et al. published a population-based case–control observational study showing a causative relationship between cyclooxygenase-2 inhibitors and SJS/TEN appearance [51]. Another study by La Grande et al. emphasized the need to increase awareness regarding the severe, life-threatening adverse reactions of COX-2 inhibitors [40]. Lastly, a recent surveillance data analysis regarding the adverse reactions reported after NSAID use highlighted some extremely important issues: among NSAID monotherapies with acetaminophen, ibuprofen, aspirin, diclofenac, and celecoxib, ibuprofen had the highest association with SJS, but the lowest fatality rate, with celecoxib having the latest onset time to SJS appearance and diclofenac being associated with the highest risk of death [52].

Considering the large consumption of painkillers, especially anti-inflammatory drugs, with or without medical prescriptions, we consider it important to reiterate the need to control this phenomenon, especially in terms of preventing fatal adverse reactions. Starting by presenting the case of our patient, we continued with an updated review of the main diagnostic and treatment features related to this severe allergic reaction of tenoxicam use.

## 2. Case Presentation

**Figure 1:**
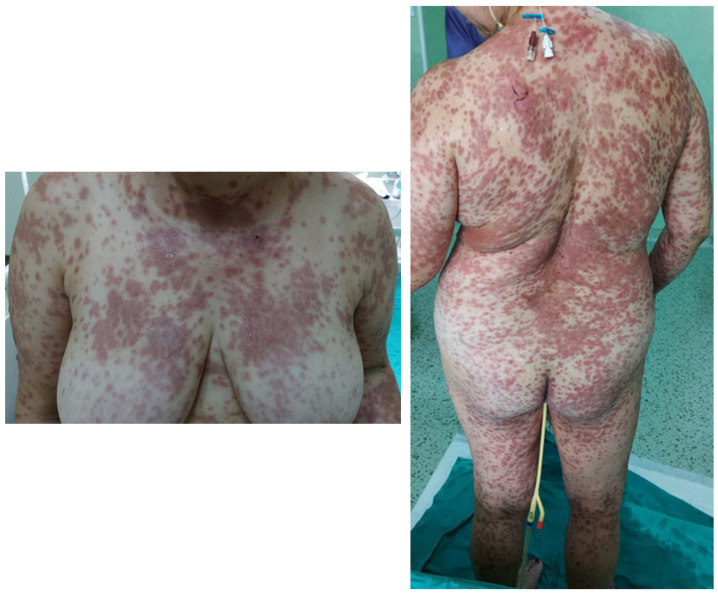
Polymorphous vesicular and bullous eruption affecting almost 90% of her total body surface area (TBSA) with areas of skin detachment.

**Figure 2:**
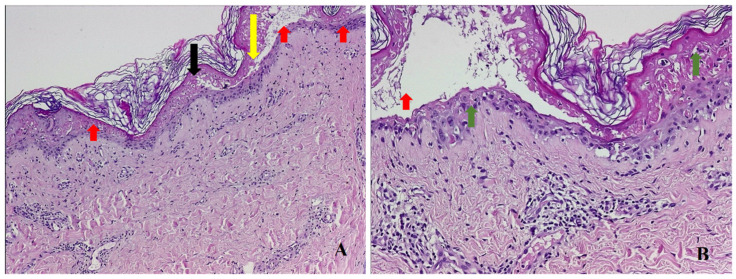
Skin biopsy—hematoxylin eosin staining showing transepidermal necrosis (black arrow) and dermis–epidermis detachment (yellow arrow), multiple inflammatory cells, and vacuolization at the dermo–epidermal junction (red arrows). (**A**) (HE, ×50); lymphocytic inflammation at the dermis level and at the dermo–epidermal junction (green arrows). (**B**) (HE, ×200).

**Figure 3:**
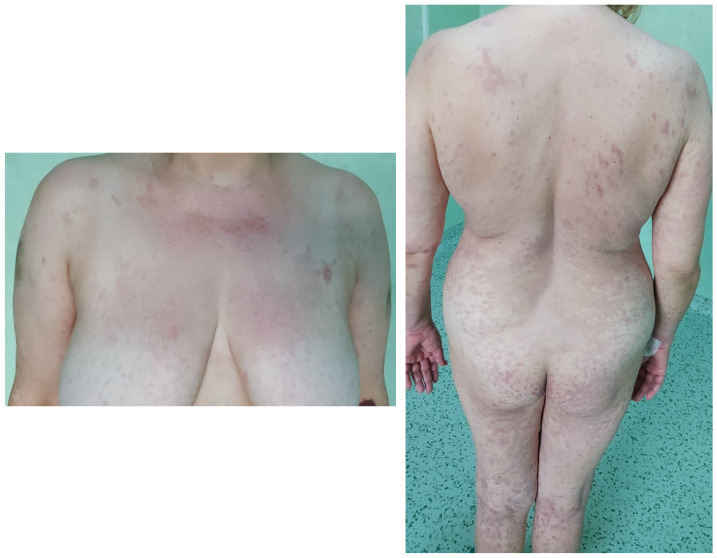
Resolution of skin lesions with persistent areas of hyperpigmentation.

A 50-year-old Caucasian female, with a history of arterial hypertension and no history of drug allergy, was referred to our hospital five days after a rash eruption on the neck and neckline and oral erosions, which progressively worsened. Ten days before presentation, she received tenoxicam 20 mg/day for lower back pain. After 5 days, she developed the aforementioned symptoms and received 16 mg of methylprednisolone twice a day and 5 mg/day of levocetirizine from her dermatologist and discontinued tenoxicam. However, the condition worsened, and she was admitted into the burn unit of the Emergency Clinical Hospital of Bucharest. She had a polymorphous vesicular and bullous eruption affecting almost 90% of her total body surface area (TBSA) (almost 10% skin detachment) (Figure 1), as well as skin erosion all over her face, hands, thighs, back, and genital region. The patient presented oral, nasal, ocular, and vaginal mucosa lesions, with mild dysphagia, severe asthenia, tachycardia (heart rate > 120 beats per minute), and a tendency for hypotension (blood pressure, 75/46 mmHg), which was fluid-responsive. The complete blood count showed leukocytosis at 11.000/uL, hyperglycemia of 165 mg/dL, hyponatremia of 130 mmol/L, hypokalemia of 3.15 mmol/L, and hypocalcemia of 0.99 mmol/L. All the other bioumoral parameters (baseline biochemistry, coagulation panel, blood urea nitrogen, creatinine, lactate, fibrinogen, albumin, total protein, alanine aminotransferase, aspartate aminotransferase, bilirubin, cholesterol, creatine kinase, and creatine kinase-MB) were within a normal range. Genetic tests could not be performed during hospitalization. The diagnosis was considered as TEN. A SCORTEN [53] score (Table 1) of 3 points was calculated. Using the algorithm for the assessment of drug causality in epidermal necrolysis (ALDEN) [54], we obtained a score of 6 points, indicating that the suspected drug was very likely the causative agent. Additionally, a Naranjo score [55] of 9 points sustained the implication of the incriminated agent. Nikolsky’s sign (tenderness of the tegument, with dislodgment of the epidermis and extension of the blisters at lateral pressure application) [22] was positive. Upon admission, systemic corticosteroids were initiated with 500 mg of methylprednisolone twice a day for 5 days, followed by tapering, along with 5 mg/day of levocetirizine, 40 mg/day of omeprazole, a replacement of fluid loss to maintain a urinary output of 0.5–1 mL/kg/hour, a correction of electrolyte imbalances, nutritional support, pain control, and general hygiene using chlorhexidine 4%. For the nasal mucosa lesions, the otorhinolaryngologist prescribed topical applications with hydrocortisone butyrate, and they prescribed topical hyaluronic acid for the oral ulcerations. The ophthalmological consultation revealed eyelid and conjunctival lesions, eye pain, and slightly blurred vision, and a topical treatment with a tobramycin/dexamethasone ophthalmic solution, along with periodic flushing with a saline solution, was initiated. The patient was evaluated by the gynecologist, who diagnosed vulvovaginitis based on an erythematous vulva and vaginal mucosa with moderate leukorrhea. A full bacterial screening was performed upon admission, identifying the presence of *Staphylococcus epidermis* in the ears, conjunctival secretions, and *Escherichia coli* in vaginal secretion. Considering that the main differential diagnosis for SJS/TEN are mainly infectious diseases, along with the increased risk of infections in face of important rapid skin detachment, the patient was evaluated by an infectious disease specialist. Due to the low SCORTEN score, along with minor leukocytosis and negative procalcitonin, there was no need for antibiotic initiation. On the second day of hospitalization, due to erosive lesions on the oral and nasal mucosa and evolving epidermal detachment (almost 30%), a skin biopsy was performed, confirming the previously established diagnosis of SJS-TEN overlap 48 h after admission to the intensive care unit. The histopathological appearance was suggestive of vesicular dermatosis with transepidermal necrolysis, which was consistent with Stevens–Johnson syndrome (Figure 2). Starting from the 10th day of intensive care, a regression of the lesions was observed, with the appearance of areas of tissue re-epithelialization. Urinary bacteriological examination revealed the presence of *Escherichia coli* and *Klebsiella pneumoniae*, both of which are sensitive to quinolones, and due to the association of dysuria and leukocytosis, the nephrologist decided to initiate antibiotic therapy with 500 mg/day of levofloxacin for 5 days. Systemic cortisone therapy was tapered from intravenous to oral administration until complete discontinuation. Under this complex management, after three weeks, the skin was fully re-epithelized, with areas of skin hyperpigmentation (Figure 3) and mild anxiety. The patient was able to be discharged with the recommendation to avoid the lifelong use of all selective COX inhibitors agents.

## 3. Discussion

### 3.1. Clinical Presentation and Diagnosis

It is well known that there are no established diagnosis criteria for SJS and TEN, even though they are among the few dermatological emergencies. The diagnosis was based on clinical features obtained during a detailed physical examination, along with the existence of a causative drug, and histological confirmation. Usually, the mucocutaneous eruption (diffuse erythema) onset coincides with a febrile episode and malaise (prodrome), followed by macula and larger bulla formation and skin detachment, which are associated with compromising the integrity of at least two mucous membranes (systemic involvement) [1,18,19,22,56,57,58]. Oral erosions and crust formation, keratoconjunctivitis, and the loss of nails or eyebrows are commonly observed. Due to keratinocytes necrosis, the epidermis’s detachment from the dermis appears (keratinocytes lose their shape and their adhesion ability), which is associated with the formation of extremely painful blisters [58,59,60].

### 3.2. Skin Biopsy and Pathophysiology

A skin biopsy may help identify a differential diagnosis, and it is recommended for severe forms [61]. There are some burn centers taking biopsies in all patients with high clinical suspicion [62,63]. In our case, the biopsy was performed considering the severe form of the syndrome and confirmed the presence of transepidermal necrolysis. The main histopathological feature in SJS/TEN skin lesions is keratinocyte apoptosis leading to epidermal necrosis, followed by dermo–epidermal disjunction [64]. Lymphocyte infiltration at the dermis level is often observed [65]. There are still controversies regarding SJS/TEN pathophysiology, with some studies leaning towards the involvement of immune-mediate reactions, including not only the innate immune system but also the adaptive one [66]. The excessive activation of CD 8+ cytotoxic T lymphocytes and natural killer (NK) cells, along with the enhanced production of granulysin (a proapoptotic protein), has been associated with keratinocyte apoptosis and disease severity [67]. Furthermore, the soluble Fas ligand (sFasL), resulting from keratinocyte or peripheral blood mononuclear cells (PBMCs), interacts with the Fas receptor on the cell membrane of keratinocytes, promoting their own apoptosis. Serum sFasL has shown potential for the early diagnosis of SJS/TEN [68]. Subsequently, keratinocyte apoptosis is extended by the activated inflammatory cascade, especially via tumor necrosis factor (TNF)-alpha and reactive oxygen species [69].

### 3.3. Differential Diagnosis

The differential diagnosis of SJS/TEN includes various infectious diseases, especially bullous pemphigoid, pemphigus vulgaris, and staphylococcal scalded skin syndrome (SSSS) [70]. Infections caused by *herpes simplex virus* and *Mycoplasma pneumoniae* can lead to oral mucositis and skin involvement but are not associated with skin detachment [16]. Other differential diagnoses include acute generalized bullous pustulosis (AGEP), autoimmune blistering diseases (paraneoplastic pemphigus), and generalized bullous fixed drug eruption (GBFDE) [56,71]. Lastly, acute graft versus host reaction may be confused with SJS/TEN, but it usually appears within 2 weeks after the cell transplant [56]. By considering the patient’s history, conducting bacteriological screening, and assessing bioumoral markers, we have excluded the main differential diagnoses.

### 3.4. Prognostic Scores

The SCORTEN score is widely used as a prognostic toll, and it is recommended to be calculated on day 1 and 3 of hospitalization [53]. It consists of seven independent variables as follows: age ≥ 40 years, cancer/malignancy presence, heart rate ≥ 120 beats per minute, body surface area detached ≥ 10% at day 1, serum glucose > 14 mmol/L, serum blood urea nitrogen > 10 mmol/L, and serum bicarbonate < 20 mmol/L. The score varies from 0 to ≥5, with mortality rates increasing with each point above 0 (3.2%, 12.1%, 35.3%, 58.3%, and >90%) [56]. A recent study has shown that this scoring system is accurate in predicting mortality when the value is high, and therefore, new prognosis scores should be used in cases with a low SCORTEN score [72]. In our case, the risk of mortality was 35.3%, but we should take into consideration that a patient with epidermal necrolysis of around 90% of the body surface area is predisposed to many complications. Therefore, a rapid, and multidisciplinary approach is always recommended in severe forms of SIS or TEN, even if the prediction scores are favorable.

The ABCD-10 (age, bicarbonate, cancer, dialysis, 10% body surface area) scale has been recommended for prognosis evaluation as an alternative for SCORTEN score, especially for patients with previous comorbidities. However, reports have shown that the ABCD-10 score could predict in-hospital mortality, especially in patients developing acute kidney injury (AKI). The SCORTEN scale should be used for predicting inpatient mortality for cases with epidermal necrolysis [73,74,75]. Sassolas et al. showed that the ALDEN algorithm, due to its sensitivity, can be used as a reference method for the assessment of drug causality in patients with SJS/TEN [54].

### 3.5. Treatment Algorithm and Multidisciplinary Approach

The algorithm treatment is not well established [76]. The main principles of treatment in SJS and TEN involve the withdrawal of the causative agent, supportive care, nutritional support, and systemic corticosteroid therapy. However, these treatments should be adapted based on the patient’s clinical status, history of comorbidities, current chronic medication, and the systemic immune and inflammatory response [16,61,77]. Plasmapheresis may be used by removing the causative agent, but the results are controversial [78,79]. Intravenous immunoglobulins show promising results for the treatment of SJS/TEN syndromes, especially in combination with systemic corticosteroids [80,81,82]. There are some reports about the usefulness of tumor necrosis factor inhibitors, calcineurin inhibitors, or combination therapies in such cases [83,84,85]. Current recommendations suggest a multi-faceted regimen, with the use of systemic corticosteroids and intravenous immunoglobulins being under continuous assessment [56]. We opted for a corticosteroid regimen, including systemic and topical applications, to inhibit the inflammatory cells and the associated cytokines [86]. Additionally, we applied topical drug solutions to all affected mucosal areas, resulting in a staged detachment of the epidermis, the progressive resolution of the mucosal ulcerations, and re-epithelization. We have not used the active debridement of the necrotic epidermis. Studies have shown that the detached epidermis acts as a biologic dressing and hastens the process of re-epithelization [34].

As we emphasized before, considering that SJS/TEN are rare diseases, there are still controversies regarding the optimum therapeutic management. An important meta-analysis published by Zimmermann et al. showed that early systemic corticosteroids, used as pulse therapy, have beneficial effects. In addition to these, promising results have also been shown by cyclosporine, especially regarding mortality. The use of intravenous immunoglobulins (Ig) is not supported by this analysis [87]. Hirahara et al. stated that using high doses of corticoids early in the disease leads to inflammation inhibition, decreasing the serum levels of interleukins [88]. Others suggested that these high doses are associated with increased risks of infections and gastrointestinal bleedings [89]. Currently, the UK guidelines do not recommend any specific active interventions except for supportive care in cases of SJS/TEN [77]. The Japanese guidelines state that treatment should start with steroids at 0.2–5 mg/kg/day as a first-line therapy, followed by intravenous Ig and plasmapheresis as last resorts in patients with severe unresponsive forms [81].

In addition to cutaneous lesions, there is frequent multiorgan involvement, especially ophthalmological, genitourinary, cardiovascular, and pulmonary. Hypoxia, pulmonary edema, pneumonia, emphysema, hepatitis, urethritis, glomerulonephritis, skin infection, vaginal synechiae, and sepsis have been reported [4,9,15,90]. In our case, the severe cutaneous reactions were associated with eye, nose, oral, and vaginal involvement, and the infectious component presented late and required multidisciplinary therapeutic management to obtain the best outcome. A study by Lim et al. conducted in a burn unit emphasized the need for specialized intensive care management due to the severity, rapid progression, and unpredictable nature of the syndromes [62]. The current United Kingdom (UK) guidelines prioritizes multidisciplinary supportive care above systemic treatment [77].

Burn units can offer a proper environment in terms of optimum fluid repletion and the prevention of infection, two of the main key aspect of patients with severe form of TEN [83]. The UK guidelines regarding the management of patients with SJS/TEN implies that cases with clinical deterioration, extensive epidermal detachment, local pus or sepsis, or delayed wounds should be referred to a burn center [77].

Due to the systemic involvement, long-term sequels are commonly seen after SJS or TEN. They include skin hyperpigmentation, hair loss, ocular problems (dry eye, chronic conjunctivitis, and foreign body sensation), oral mucosa problems, scarring, anxiety, depression, post-traumatic stress disorder, and the inability to resume daily work [91]. Some patients require transfer to a rehabilitation center before returning home or require a caregiver. All these factors contribute to an impaired quality of life. For patients with conjunctival involvement, long-term complications are dry eye syndrome, photophobia, ectropion, corneal abrasions or ulcers, lid adhesions, and the loss of visual acuity [63,92]. As for patients with vulvovaginal involvement, the main reported complications are synechiae formation and vaginal stenosis, requiring surgery [63,93]. The appearance of hypertrophic scars is extremely rare [94]. Fortunately, in our case, the only long-term complications were skin hyperpigmentation, which we believe will correct in time with proper management and avoidance of sun exposure, and mild anxiety. All the above stresses the importance of the long-term follow-up of these patients in order to promptly treat complications to reduce further morbidity.

## 4. Conclusions

Allergies to common pain medication are becoming more frequent due to the abuse of painkillers; therefore, actions must be taken to stop this phenomenon and to increase vigilance for adverse reactions. The present case report strengthens the need for a multidisciplinary approach in patients presenting with severe toxic epidermal necrolysis and multiorgan involvement. The immediate cessation of the incriminated drug; the rapid initiation of systemic steroids, along with topical therapies; and isolation into a specific environmental condition are of paramount importance to prevent mortality and to decrease morbidity. Nevertheless, we reiterate that severe or complicated cases should be transferred to a specialized burn center.

## Figures and Tables

**Table 1:** The patient’s SCORTEN score.

Parameter	Points
age ≥ 40 years	1
cancer/malignancy	0
heart rate ≥ 120 beats per minute	1
body surface area detached ≥ 10% at day 1	1
serum glucose > 14 mmol/L	0
serum blood urea nitrogen > 10 mmol/L	0
serum bicarbonate < 20 mmol/L	0
Total Value	3 points
Mortality Risk	35%

## Data Availability

Research data are available, upon reasonable request, to the corresponding authors.
